# Light Scattering and Rheological Studies of 3D/4D Printable Shape Memory Gels Based on Poly (*N*,*N*-Dimethylacrylamide-co-Stearyl Acrylate and/or Lauryl Acrylates)

**DOI:** 10.3390/polym13010128

**Published:** 2020-12-30

**Authors:** MD Nahin Islam Shiblee, Kumkum Ahmed, Yuta Yamazaki, Masaru Kawakami, Hidemitsu Furukawa

**Affiliations:** 1 Department of Mechanical Systems Engineering, Graduate School of Science and Engineering, Yamagata University, Yonezawa, Yamagata 992-8510, Japan; tfh41312@st.yamagata-u.ac.jp (Y.Y.); kmasaru@yz.yamagata-u.ac.jp (M.K.); furukawa@yz.yamagata-u.ac.jp (H.F.); 2 Shibaura Institute of Technology, Koto City, Tokyo 135-8548, Japan

**Keywords:** shape memory polymers, 3D/4D printing, light scattering, rheology

## Abstract

In this work, we present the structural analysis of 3D/4D printable *N*,*N*-dimethylacrylamide (DMAAm)-co-stearyl acrylate (SA) and/or lauryl acrylate (LA)-based shape memory gels (SMGs). We characterized these gels by scanning microscopic light scattering technique (SMILS) where a time- and space-averaged correlation function is obtained to overcome the inhomogeneous media. Thus, the characteristic size of the gel internal network (mesh size, ξ) and crosslinking densities are estimated from the Einstein–Stokes formula. The rheological study of the SMGs revealed information about their mechanical strength and transition temperature. From the experimental storage modulus measured by rheological study, crosslinking density and mesh size of the network were also calculated. Both the techniques suggest that SMG with high crystalline content of SA monomer in the gel network contain smaller mesh size (1.13 nm for SMILS and 9.5 nm for rheology study) and high crosslinking density. The comparative study between the light scattering technique and rheological analysis through the quantitative analysis of crosslinking densities will be important to understand the structural properties of the SMGs.

## 1. Introduction

**Figure 1:**
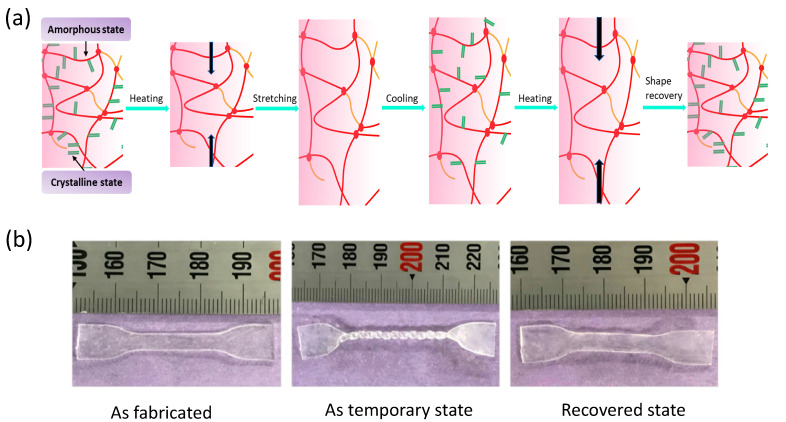
(**a**) Illustration on shape memory property of shape memory gel (SMG), (**b**) demonstration of shape memory behavior of 3D printed SMGs.

Smart functional materials based on softness, flexibility, and wet nature are showing numerous prospects in material science, engineering, and biomedical sectors [1,2,3,4,5]. Shape memory gel (SMG) materials are one of the most promising materials that have the potential to realize the future demand as intelligent materials owing to their several distinctive characteristics such as excellent shape fixity, outstanding shape recovery, tunable elasticity, and swelling properties [6,7,8]. Freedom of designability with rapid prototyping capability has gained significant importance among the engineers and materials scientists for the utilization of such smart and functional materials in task-specific applications such as in the development of soft actuators and sensors. Thus, 3D printing, or additive manufacturing, is gaining significant attention and already pushing the boundaries towards the 3D fabrication of soft polymeric materials in diverse areas including biomedical, energy, and soft robotics [1,2,3,4,8]. Our research group has successfully developed various types of 3D printable functional gels and composite materials such as ionic liquid (IL)-based nanocomposites, ionic gels, and shape memory gels (SMGs) [7,8,9,10,11,12]. Among them, 3D and 4D printable SMGs prepared by copolymerization of hydrophilic monomer *N*,*N*-dimethylacrylamide (DMAAm), and crystalline hydrophobic monomer stearyl acrylate (SA) and/or lauryl acrylate (LA) offer fascinating features of shape memory effect that are highly desirable properties for implementing them in applied sectors. 3D and 4D printability facilitated their applications in the soft robotics and actuator fields by overcoming the problems associated with molding and shaping. As demonstrated in Figure 1a, the shape memory effect of SMGs is governed by the melting and recrystallization of SA and LA monomers. Thus, the shape of SMGs can be repeatedly exchanged between the original shape and a temporary deformed state Figure 1b. A detailed study on the 3D and 4D printability of SMGs with soft robotic functions has already been reported previously [7,8]. In this work, we present the structural properties and rheological properties of DMAAM and SA/LA-based SMGs to understand their internal network structure and viscoelastic properties, which we believe, will be important to understand such materials more intensively and enable better tuning of the physico-chemical properties. Theoretically, mesh size or in other words distance between crosslinkers in gels is calculated by tree-like approximation and real space renormalized effective medium approximation while experimentally, many different sizes and methods have been proposed to estimate mesh sizes such as correlation blob by scattering experiments [13,14], elastic blob by rheological measurements [13,15,16], and mesh-like structure observed in scanning transmittance electron microscopes [17,18]. Dynamic light scattering (DLS) is one of the most popular methods to study the chemical gelation of different polymer systems, e.g., poly(*N*-isopropylacrylamide) [8], poly(methyl methacrylate) [9,10,11], poly(dimethylsiloxane) gels [12], and other randomly branched polymer systems [13] have been previously studied [19,20,21,22,23]. The scanning microscopic light scattering (SMILS) method has been established to be one of the very effective ways to nondestructively characterize the internal structure of solid gels [24,25,26,27]. The SMILS is a typical dynamic light scattering (DLS) system, which characterizes the internal structure of gels by observing the diffusion process. In our previous studies, we studied internal structures of poly (*N*-*N*-dimethylacrylamide (PDMAAm) gels and different types of inter-crosslinking network (ICN) gels, ionic gels, and end-crosslinked gels via the technique of SMILS. The information about the mesh size, crosslinking density, and diffusion behavior of the gels are advantageous for different applications such as immobilization of dyes or enzymes or entrapping specific molecules in a gel network. Using this method, we can also explain useful information on the properties of the network structure in the gels, such as mesh-size, defects in network, concentration fluctuation, i.e., inhomogeneities, and so on. On the other hand, rheological study can provide bulk mechanical properties along with crosslinking densities of polymer networks. In this work, for the first time, we have utilized the SMILS technique and rheology to observe the network structure of 3D printable and healable SMGs. Mesh chain densities or in other words crosslinking densities of the gels have been estimated from the mesh size of internal structure calculated by SMILS as well as from the experimental storage modulus from the rheology study. Rheological properties also reveal the effect of crystalline monomer content on the mechanical properties and transition temperatures. The comparative study on mesh size and crosslinking densities obtained by each method will help better understand the internal structure of these multifunctional gels.

## 2. Materials and Methods

### 2.1. Materials

Materials for SMG synthesis: Hydrophilic monomer DMAAm was purchased from Tokyo Chemical Industry Co. Ltd., Tokyo, Japan. Crystalline hydrophobic monomer SA and LA, crosslinker *N*-*N*’ methylenbisacrylamide (MBAA), and initiator α-keto glutaric acid (α-keto) were purchased from Wako Pure Chemical Industries, Ltd., Osaka, Japan. Initiator Diphenyl(2,4,6-trimethylbenzoyl)phosphine oxide (TPO) was purchased from Sigma Aldrich, St. Louis, MO, USA. UV absorber 5-benzoyl-4-hydroxy-2-methoxybenzenesulfonic acid (Kemisorp 11S) and Benzenesulfonic acid,2,2′-(1,2-ethenediyl) bis[5-[[4-methoxy-6-(phenylamino)-1,3,5-triazin-2-yl] amino], sodium salt (1:2) (AS150) were purchased from Sigma-Aldrich and Nippon Kagaku Co. Ltd., Tokyo, Japan.

### 2.2. Preparation of SMG Samples

SMG Synthesis process: 3D printing was done by a customized stereolithographic 3D printer named Soft and Wet Industrial Materials-Easy Realizer (SWIM-ER) and a low-cost commercial LCD 3D printer called Phrozen Shuffle. The detailed process of SMGs via 3D printing is described in the previous studies [7,8]. Briefly, gel solutions for printing have been prepared by mixing monomers, crosslinker, initiator, and UV absorber respectively to a particular ratio. Three different compositions have been prepared varying DMAAm, SA, and LA content. DMAAm compositions of the three gels are 0.75 M, 0.75 M, and 0.80 M while SA contents are 0.20 M, 0.05 M, and 0.15 M, respectively. The remaining content is LA i.e., 0.05 M, 0.20 M, and 0.05 M, respectively. Crosslinker and initiator (α-keto for SMILS and TPO for Phrozen Shuffle) were added at a 0.05 mol% and 0.6 mol% and 0.56 mol%) respectively. Finally, UV absorber (Kemisorp 11S for SMILS and AS150 for Phrozen Shuffle) was added at a 0.05 wt%. The gels are termed as SMG75-SA20-LA5, SMG75-SA5-LA20, SMG80-SA15-LA where the number followed by SMG, SA, and LA refer to the DMAAm, SA, and LA content multiplied by 100, respectively. Prior to printing, the gel solutions have been stirred for 15 min at 60 °C with a continuous supply of N2 gas to create an inert environment. Then it was printed using SWIM-ER and/or Phrozen Shuffle.

### 2.3. Method of Characterization

To understand the internal structure of the SMGs samples, light scattering experiments were carried out by SMILS using the same method as mentioned elsewhere [23,24,25]. Briefly, a laser source with a wavelength of 532 nm (manufactured by Laser Quantum Co. Ltd., Stockport, UK) was used as a light source for the scattering experiments. A rectangular-shaped SMG specimen was cut from a 3D printed SMG sheet (printed by SWIM-ER) followed by placing the sample test tube which was then set in the sample holder of SMILS. The sample test tube was then filled with water and waited for at least 24 h to allow swelling. Unless mentioned otherwise the measurement temperature was set at 30 °C. The scattering angles were chosen in the range between 40–125°. To calculate the ensemble average, 31 different positions of gels have been scanned. Hence, we can obtain a time and space-averaged correlation function, i.e., an ensemble-averaged correlation function to overcome the non-ergodicity of inhomogeneous media. The rheological properties of different hydrogels were investigated by Rotational Rheometer (MCR 302 Modular compact Rheometer) (Anton Paar, Tokyo Japan) with a 25 mm parallel plate (PP25/P2). Dynamic strain sweep was performed in the strain amplitude range of 0.0001–10% at a fixed frequency of 0.63 rad/s. Dynamic temperature sweep was performed at the heating ramp from 0 °C to 80 °C at a fixed strain of 1% and angular frequency of 5 rad/s.

## 3. Results and Discussion

### 3.1. 3D/4D Printability of SMGs

**Figure 2:**
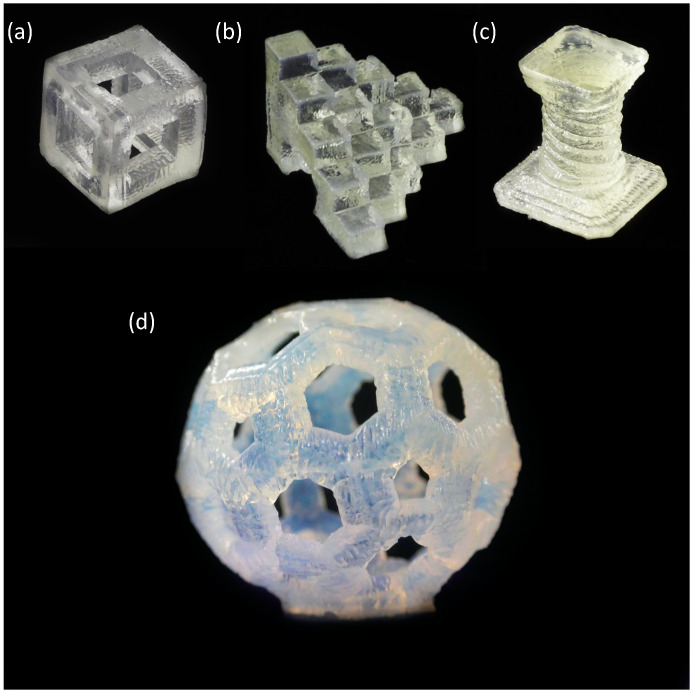
3D printed SMG structures (**a**) Koji cube (20 mm^3^ outer dimensions with a 10 mm^3^ hollow inside) (**b**) 5 mm calibration cube step (**c**) spiral pillar (34 mm height, bottom stage 30 mm^2^ and upper stage 21 mm^2^) (**d**) Buckyball (outer diameter 34.5 mm); Material: SMG80SA15LA5.

In the previous work, we reported the 3D and 4D printability of SMGs in detail with prospective applications in soft robotics using a sophisticated customized stereolithographic 3D printer named SWIM-ER [7,8]. A video demonstration on the shape memory property of a 3D printed Koji cube is added as Supplementary Information. To confirm the universal usability of the SMGs, here we demonstrated the 3D printability of SMGs via a low-cost commercial LCD 3D printer (Phrozen Shuffle printer). A variety of shapes and structures were successfully printed with good printing resolutions. Figure 2 shows various printed SMG structures using Phrozen Shuffle printer e.g., Koji cube, 5 mm calibration cube step, spiral pillar, and buckyball. This demonstration shows the facile fabrication process of these gels which, we believe, will facilitate better utilization of these materials in near future.

### 3.2. Demonstration of Shape Memory Assisted Healing Properties

**Figure 3:**
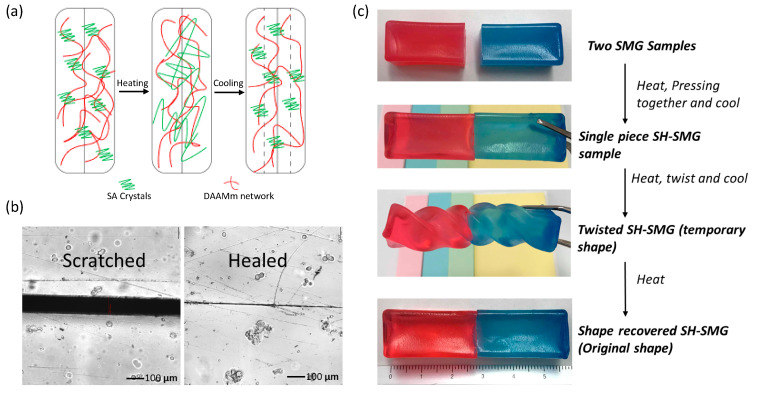
(**a**) Illustration of the self-healing mechanism, (**b**) micrographs of scratched and healed surface, and (**c**) demonstration of self-healing and shape memory characteristics.

Similar to the characteristics of shape memory polymers, the shape memory behavior of SMG can be used to repair cracks, allowing the two polymer surfaces to diffuse and heal intrinsically upon heating. Figure 3a shows the healing process of SMGs where a cracked or cut surface is healed upon heating above the transition temperature of the SMGs through melting and recrystallization of crystalline/semicrystalline SA/LA content. Figure 3b exhibits the micrographs of the cracked and healed surface while a demonstration of combined self-healing and shape memory characteristics of SMGs is presented in Figure 3c. In Figure 3b, left side of the figure shows a damaged surface of SMG where ~150 μm gap was created due to scratching while the right side of the figure shows that the scratching was minimized to a considerable extent just by heating the damaged sample at 60 °C for 5 min. No external pressure was added during this time. This type of physical healing was possibly associated with the shape memory effect of the SMG network.

### 3.3. Mesh Size and Crosslinking Densities of SMGs Calculated by SMILS

Using the SMILS technique, it is possible to successively scan the samples at different positions in a vertical direction in the micrometer scale. Thus, it is possible to overcome the inhomogeneous media in gels by obtaining a time and space-averaged correlation function i.e., an ensemble-averaged correlation function. For each sample, the time-averaged homodyne correlation functions were determined at several points, and then the ensemble-averaged correlation function g^(1)^_en_ (q,τ) (where q is scattering vector and τ is correlation time) was calculated. The detailed theory is described elsewhere [24,25,26]. Thus, the characteristic size of diffusing objects (mesh size) ξ is estimated from the Einstein–Stokes formula as given below:

D = k_B_T/3πηξ (1)

where D is the diffusion coefficient, k_B_ is the Boltzmann constant, T is the absolute temperature and η is the viscosity of the solvent.

In the measurements of SMG samples, we selected the angle based on the results obtained from the relaxation distribution function. Due to the structural inhomogeneity in the gels, a suitable angle was experimentally determined.

**Figure 4:**
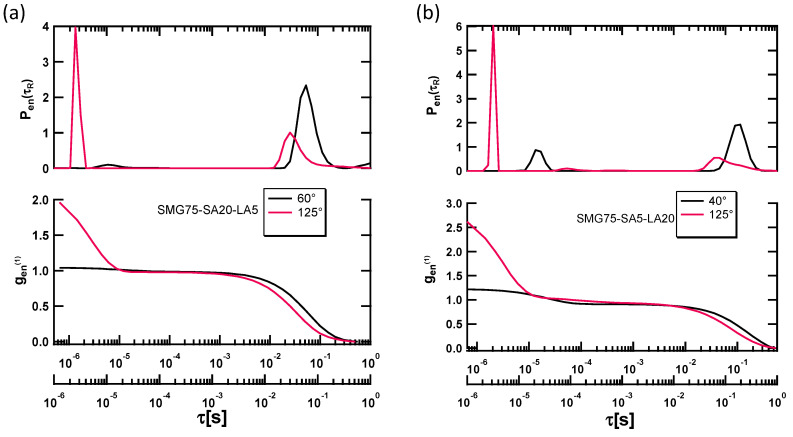
Dynamic Light Scattering analysis using SMILS of SMGs at 30 °C (**a**) Scattering angle-dependence autocorrelation function and relaxation distribution function of SMG75-SA20-LA5 as a function of the relaxation time. (**b**) Scattering angle-dependence autocorrelation function and relaxation distribution function of SMG75-SA5-LA20 as a function of the relaxation time.

Figure 4a,b represents information about scattering angle-dependence autocorrelation function and scattering angle-dependence relaxation distribution function of SMG75SA20LA5 and SMG75SA5LA20. From these figures, it is clearly understood that the density probability P_en_ (τ_R_) depends on the scattering angle. The small relaxation time indicates a smaller mesh size. The fast mode of the relaxation time has been considered due to the mesh motion and peak for distribution function has been found to be shifted to the right with a change in scattering angle. The diffusion coefficient, D was calculated from the slope of the straight line of the graph of the inverse of relaxation time of the Brownian motion, as a function of the square scattering vector (Supplementary Figure S1). The mesh size was calculated using Equation (1) and the crosslinking density has been calculated (ν_S_) by the following Equation (2).

ν_s_ = 1/ξ_s_^3^ (2)

where ξ_s_ denotes mesh size of the gel network. Results for SMG80-SA15-LA5 have been shown in Supplementary Figure S2. Information of the network structure is shown in Table 1.

It was observed that the mesh size of SMG is highest when crystalline SA content is lowest in the gel system which might be related to the higher crystallinity of SA monomer. As reported earlier, [25,26] pure PDMAAm gel has a mesh size of 8 nm that indicates that the co-monomer SA and LA enhanced the crosslinking density within the gel network. Therefore, it can be said that by changing the crystalline monomer content we can modulate the mesh size of the polymer network. To verify this finding in the following section, we estimated the crosslinking densities of the gels from the rheology study of the gels in the following section.

### 3.4. Rheology Study and Estimation of Crosslinking Density and Mesh Size of SMGs

Using SMG-based hydrogels as biomaterials, it is important to understand the structural parameters, e.g., stiffness, mesh size, crosslinking density of the polymer chain. Rheology study is another powerful tool to understand these structural parameters of gel material. Through the rheological study, we investigated the stiffness, transition temperature, and mesh size.

**Figure 5:**
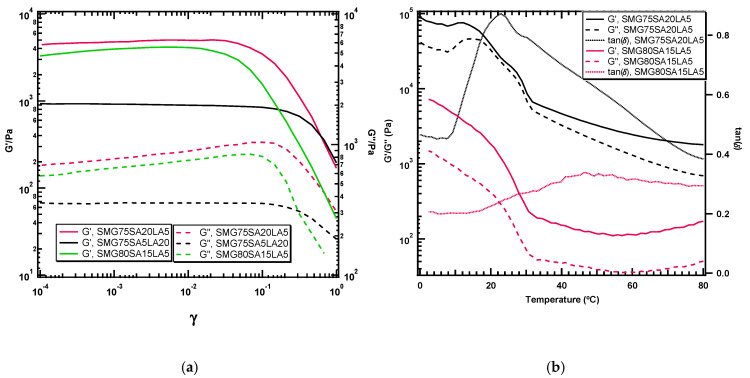
(**a**) The storage (G′) and loss (G″) modulus at 10 rad/s for SMG samples as a function of the applied strain (**b**) Temperature-sweep analysis of the gel samples.

Oscillatory rheology measurements were performed with varying deformation amplitude at a fixed frequency (strain sweeps) to determine the linear viscoelastic region of each sample. The results of amplitude sweeps are presented in Figure 5a which provided the information on the effect of crystalline network SA and LA content on the mechanical strength of SMGs. It is observed that for all the SMG samples, both the G′ and G″ curve exhibited nearly plateau points with different levels. For all the SMG samples, the elastic modulus G′ is dominating the viscous modulus which confirmed the gel-like texture. The mechanical strength of the SMG samples is evaluated by comparing the values of G′ in the linear viscoelastic region. SMG75-SA5-LA20 exhibited the lowest G′ value while SMG75-SA20-LA5 has the highest G′ value owing to the lowest and highest content of crystalline SA content in the gel network respectively. By controlling the composition of hydrophobic monomer LA and SA in the copolymer systems, SMGs with variable mechanical stiffness can be synthesized. The crystalline SA tends to make the SMG more rigid while LA contributes to the flexibility and make the SMG soft. This behavior well justified the results of mechanical properties (tensile tests) reported previously [7].

The influence of temperature on structural change and information about the transition temperature of the SMG samples were determined by temperature sweep in the temperature range 0–80 °C. The G′, G″, and loss factor (tanδ) of the SMG75-SA20-LA5 and SMG80-SA15-LA5 with different compositions against temperature are plotted in Figure 5b. Distinguishable changes on the storage and loss modulus occurred over 20 °C for all the samples while no overlapping between G′ and G″ (tanδ < 1) ensured the dominance of the elastic region over the whole temperature range. It can be noted here that SMG75-SA20-LA5 exhibited sharp tand value while tanδ values of SMG75-SA20-L5 and SMG80-SA15-LA5 are rather broad over a wide temperature range. This phenomenon can be described by the presence of amorphous network DMAAm and less crystalline LA in the SMG samples in higher content resulting in gel samples with an amorphous nature [7]. The crosslinking densities of the SMGs have been calculated from the G′ values of rheology experiments (strain sweep). The crosslinking density and average mesh size (ξ, nm), which is defined as the distance between the crosslinking points, can be calculated based on the rubber elastic theory from the following equations [28,29]

ν_R_ = (G′N_A_/RT) (3)

where G′ is the storage modulus, N_A_ is the Avogadro constant (6.022 × 10^23^), R is the gas constant (8.314 J/K mol) and T is the temperature (303 K).

ν_R_ = 1/ξ_R_^3^ (4)

where ξ_R_ is the mesh size.

Table 2 presents the quantitative values of experimental crosslinking density and subsequent mesh size calculated from the storage modulus of rheology experiments. From the E values, the increasing order is SMG75-SA20-LA5 > SMG80-SA15-LA5 > SMG75-SA5-LA20 which can be well-coordinated with the presence of a higher content of highly crystalline monomer SA in the polymeric network. The result well-justified the mechanical results reported previously [7]. The calculated crosslinking densities and mesh size is listed in Table 2.

Calculated crosslinking densities from rheology experiments show a similar sequence to the values calculated by SMILS i.e., SMG75-SA20-LA5 (with highest SA content) exhibited the highest crosslinking density and SMG75-SA5-LA20 showed the lowest crosslinking densities. Although the magnitude of crosslinking densities estimated by the two methods are not exactly the same due to the difference in assumptions in the two methods. Crosslinking densities (ν_s_) using SMILS exhibited higher values for all the samples than the crosslinking densities (ν_R_) determined by rheology. ν_s_ exhibits sensitivity for minute structure related to the Brownian motion in the gel network while ν_R_ is more inclined to the macroscopic structure related to the modulus. Therefore, a lower value for ν_s_ is understandable. However, it can be noted that both the method provided similar trends on the mesh size of the SMGs. Therefore, it can be said that the internal structure of SMGs calculated by the SMILS apparatus offered an analogous method to the structural information estimated by the rheological characterization. However, it should be noted that while SMILS offers a nondestructive way of analysis while the rheological study is more suitable for turbid gel materials. Our future study will utilize the structural information for the development of 3D gel scanners which will provide the structural and mechanical information of printed gel models.

## 4. Conclusions

Infernal structural information of a series of 3D printable SMG materials has been investigated by light scattering method (using SMILS apparatus) and rheology study. Using the SMILS technique, mesh sizes, ξ of the SMGs are estimated from the Einstein–Stokes formula from which the crosslinking densities were calculated. The experimental storage modulus of strain sweep was utilized in calculating the crosslinking density and mesh size of the gel network following rubber elastic theory. Temperature sweep of rheology study also provided information on the transition temperature. The comparative study between the light scattering technique and rheological analysis through the quantitative analysis of crosslinking densities followed similar patterns which will be important to comprehend the fundamental properties of the SMGs. This study will help to understand the internal structures of these smart materials in a nondestructive way of light scattering and a comparative study between SMILS technique and rheology will suggest a better understanding of the structural properties due to the change in crystalline contents in SMGs.

## Figures and Tables

**Table 1:** Internal network information of the SMGs.

Gel Systems	Diffusion Coefficient, D	Mesh Size, ξ_s_ (nm)	Crosslinking Density, ν_s_ (m^−3^)
SMG75-SA20-LA5	4.92 × 10^−10^	1.13	6.93 × 10^26^
SMG75-SA5-LA20	3.97 × 10^−10^	1.40	3.64 × 10^26^
SMG80-SA15-LA5	4.11 × 10^−10^	1.35	4.06 × 10^26^

**Table 2:** Mechanical properties and crosslinking densities of the SMGs.

Gel Systems	Storage Modulus, G’ (Pa)	Crosslinking Density, ν_R_ (m^−3^)	Mesh Size ξ_R_
SMG75-SA20-LA5	4922	1.17 × 10^24^	9.5
SMG75-SA5-LA20	926	2.21 × 10^23^	16.5
SMG80-SA15-LA5	3759	8.98 × 10^23^	10.3

## Data Availability

The data presented in this study are available in this article and Supplementary Material.

## Supplementary Materials

The following are available online at https://www.mdpi.com/2073-4360/13/1/128/s1, Figure S1: Relaxation time as a function of square of scattering vector for determination of diffusion coefficient(a) SMG75-SA20-LA5 (b) SMG75-SA5-LA20, Figure S2: Dynamic Light Scattering analysis using SMILS of SMG80-SA15-LA5 at 30 °C (a) Scattering angle-dependence autocorrelation function and relaxation distribution function as a function of the relaxation time. (b) Scattering an-gle-dependence autocorrelation function and relaxation distribution function as a function of the relaxation time (c) Relaxation time as a function of square of scattering vector, Video S1: Demon-stration of shape memory behavior of 3D printed SMG.
