# Supplementary material for: Light Scattering and Rheological Studies of 3D/4D Printable Shape Memory Gels Based on Poly (N,N-Dimethylacrylamide-co-Stearyl Acrylate and/or Lauryl Acrylates)
Source: Polymers (Basel). 2020 Dec 30;13(1):128. doi: 10.3390/polym13010128 (PMC7794971; doi:10.3390/polym13010128)
Supplement: Supplementary file 1 [file polymers-13-00128-s001.zip › polymers-1052708-supplementary/polymers-1052708-supplementary.docx]

Supplementary Figures

Figure S1. Relaxation time as a function of square of scattering vector for determination of diffusion coefficient(a) SMG75-SA20-LA5 (b) SMG75-SA5-LA20

Figure S2. Dynamic Light Scattering analysis using SMILS of SMG80-SA15-LA5 at 30 °C (a) Scattering angle-dependence autocorrelation function and relaxation distribution function as a function of the relaxation time. (b) Scattering angle-dependence autocorrelation function and relaxation distribution function as a function of the relaxation time (c) Relaxation time as a function of square of scattering vector.

Video S1: Demon-stration of shape memory behavior of 3D printed SMG
